# Framework for responsible sharing of genomic and health-related data

**DOI:** 10.1186/s11568-014-0003-1

**Published:** 2014-10-17

**Authors:** Bartha Maria Knoppers

**Affiliations:** Global Alliance for Genomics and Health, Toronto, Canada

## Preamble

The sharing of genomic and health-related data for biomedical research is of key importance in ensuring continued progress in our understanding of human health and wellbeing. The challenges raised by international, collaborative research require a principled but nevertheless practical Framework that brings together regulators, funders, patient groups, information technologists, industry, publishers, and research consortia to share principles about data exchange. Such a Framework will facilitate responsible research conduct.

This Framework is developed under the auspices of the Global Alliance for Genomics and Health. Its mission is to accelerate progress in human health by helping to establish a common Framework of harmonized approaches to enable effective and responsible sharing of genomic and clinical data and to catalyze data sharing projects that drive and demonstrate the value of data sharing.

This Framework provides guidance for the responsible sharing of human genomic and health-related data, including personal health data and other types of data that may have predictive power in relation to health. In particular, it highlights, and is guided by, Article 27 of the 1948 *Universal Declaration of Human Rights*. Article 27 guarantees the rights of every individual in the world “*to share in scientific advancement and its benefits*” (including to freely engage in responsible scientific inquiry), and at the same time “*to the protection of the moral and material interests resulting from any scientific*…*production of which* [*a person*] *is the author*”. (As set out in Appendix 1, many other international conventions and national laws, regulations, codes and policies also guide responsible data sharing behavior).

This Framework is guided by the human rights of privacy, non-discrimination and procedural fairness. At the same time, it considers all human rights principles relevant, complementary and interrelated, founded as they are on respect for human dignity. Since science proceeds only with the broad support of society, respect for all persons is a primary driver underlying all other derived principles. In particular, this Framework establishes a set of foundational principles for responsible research conduct and oversight of research data systems in the realm of genomic and health-related data sharing. It interprets the right of all people to share in the benefits of scientific progress and its applications as being the duty of data producers and users to engage in responsible scientific inquiry and to access and share genomic and health-related data across the translation continuum, from basic research through practical applications. It recognizes the rights of data producers and users to be recognized for their contributions to research, balanced by the rights of those who donate their data. In addition to being founded on the right of all citizens in all countries to the benefits of the advancements of science, and on the right of attribution of scientists, it also reinforces the right of scientific freedom.

The value of this Framework is that it: offers political and legal dimensions that reach beyond the moral appeals of bioethics and provides a more robust governance framework for genomic and health-related data sharing; speaks to groups and institutions, not just individuals; stresses the progressive realization of duties; and urges action by governments, industry, funders, publishers, and researchers to create an international environment for responsibly sharing data.

This Framework will be elaborated by subsequent Policies (Appendix 2) on particular issues such as ethical governance, consent, privacy and security. The Framework and its subsequent Policies should be used in projects around the world (whether Global Alliance “inspired” or not) such that they become the tools that approval entities, recognized by different jurisdictions, will turn or refer to for guidance. Recognizing diversity of legal and ethical approaches and being responsive to emerging issues, both this Framework and its Policies are intended to provide leadership in this domain for wider discussion.

## Purpose and interpretation

### 1. Purpose

The purpose of this Framework is to provide a principled and practical framework for the responsible sharing of genomic and health-related data. Its primary goals are to:

i. Protect and promote the welfare, rights, and interests of individuals from around the world in genomic and health-related data sharing, particularly those who contribute their data for biomedical research;
ii. Complement laws and regulations on privacy and personal data protection, as well as policies and codes of conduct for the ethical governance of research;
iii. Foster responsible data sharing and oversight of research data systems;
iv. Establish a framework for greater international data sharing, collaboration and good governance;
v. Serve as a dynamic instrument that can respond to future developments in the science, technology, and practices of genomic and health-related data sharing;
vi. Serve as a tool for the evaluation of responsible research by research ethics committees and data access committees; and
vii. Provide overarching principles to be respected in developing legally-binding tools such as data access agreements.

### 2. Interpretation

Without ascribing legal meaning, this Framework should be interpreted in good faith and is to be understood as a whole. The Foundational Principles and Core Elements are to be understood as complementary and interrelated, as appropriate and relevant in different contexts, countries and cultures. This Framework will be supported by Policies for guidance in particular issues such as, but not limited to, ethical governance, privacy and security, and consent. For the purposes of this Framework, “data sharing” includes data transfer or data exchange between data users, or where data are made available to secondary researchers, either openly or under specified access conditions.

## Application

This Framework is intended for all entities or individuals providing, storing, accessing, managing or otherwise using genomic and health-related data, including data donors, users, and producers. This includes, but is not limited to, researchers, research participants and patient communities, publishers, research funding agencies, data protection authorities, hospitals, research ethics committees, industry, ministries of health, and public health organizations.

## Foundational principles

The Foundational Principles of this Framework guide the responsible sharing of genomic and health-related data. They also facilitate compliance with the obligations and norms set by international and national law and policies.

### Foundational principles for responsible sharing of genomic and health-related data

- Respect Individuals, Families and Communities
- Advance Research and Scientific Knowledge
- Promote Health, Wellbeing and the Fair Distribution of Benefits
- Foster Trust, Integrity and Reciprocity

## Core elements of responsible data sharing

It is good practice for those involved in genomic and health-related data sharing to have core elements of responsible data sharing in place. The following Core Elements of the Framework aid in the interpretation of the Foundational Principles to individuals and organizations involved in the sharing of genomic and health-related data. The Core Elements should be interpreted in a proportionate manner that acknowledges different levels of risk and community cultural practices. **This Framework applies to use of data that have been consented to by donors** (**or their legal representatives**) **and**/**or approved for use by competent bodies or institutions in compliance with national and international laws**, **general ethical principles**, **and best practice standards that respect restrictions on downstream uses.** Endorsement of the Framework does not preclude the development of particular guidance via Policies for specific populations (e.g. children) or issues (e.g. ethical governance, privacy and security, and consent).

### Core elements of responsible data sharing

#### Transparency

- Develop clearly defined and accessible information on the purposes, processes, procedures and governance frameworks for data sharing. Such information should be presented in a way that is understandable and accessible in both digital and non-digital formats.
- Provide clear information on the purpose, collection, use and exchange of genomic and health-related data, including, but not limited to: data transfer to third parties; international transfer of data; terms of access; duration of data storage; identifiability of individuals and data and limits to anonymity or confidentiality of data; communication of results to individuals and/or groups; oversight of downstream uses of data; commercial involvement; proprietary claims; and processes of withdrawal from data sharing.
- Implement procedures for fairly determining requests for data access and/or exchange.

#### Accountability

- Put in place systems for data sharing that respect this Framework.
- Track the chain of data access and/or exchange to its source.
- Develop processes to identify and manage conflicts of interest.
- Implement mechanisms for handling complaints related to data misuse; for identifying, reporting and managing breaches; and for instituting appropriate sanctions.

#### Engagement

- Develop mechanisms to enable citizens to make meaningful contributions to biomedical research and to partake in deliberation on how these contributions can be respected.
- Facilitate deliberation about the wider societal implications of genomic and health-related data sharing among all stakeholders, especially citizens.

#### Data quality and security

- Store and process the data collected, used and transferred in a way that is accurate, verifiable, unbiased, proportionate, and current, so as to enhance their interoperability and replicability and also preserve their long-term searchability and integrity.
- Ensure feedback mechanisms on the utility, quality, security, and accuracy of data, and their annotations, with a view to improving quality and interoperability and appropriate re-use by others.
- Establish proportionate data security measures that mitigate the risk of unauthorized access, data loss and misuse.
- Understand the issues related to lawful requests for data based on law enforcement, public health, or national security concerns.

#### Privacy, data protection and confidentiality

- Comply with applicable privacy and data protection regulations at every stage of data sharing, and be in a position to provide assurances to citizens that confidentiality and privacy are appropriately protected when data are collected, stored, processed, and exchanged. Privacy and data protection safeguards should be proportionate to the nature and use of the data, whether identifiable, coded or anonymized.
- Forego any attempt to re-identify anonymized data unless where expressly authorized by law.

#### Risk-benefit analysis

- Consider the realistic harms and benefits of data sharing on and with individuals, families and communities, including opportunity costs associated with both sharing and not sharing data. Potential realistic benefits may include development of new scientific knowledge and applications, enhanced efficiency, reproducibility and safety of research projects or processes, and more informed decisions about health care. Potential realistic harms may include invasions of privacy or breach of confidentiality and invalid conclusions about research projects.
- Conduct data sharing with a view towards minimizing harms and maximizing benefits to not just those who contribute their data, but also to society and health care systems as a whole, particularly where data pertains to disadvantaged people. Benefits arising from data sharing may not be uniformly distributed throughout communities around the world and may depend on the usability of data within a specified context, national priorities, as well as a specific community’s concern about health and interpretation of wellbeing.
- Undertake a proportionate assessment of the benefits and risks of harm in data sharing, which is periodically monitored according to the reasonable foreseeability of such harms and benefits. Such an assessment may also incorporate mechanisms that track subsequent harms, should they materialize, so as to help inform future policy.

#### Recognition and attribution

- Design systems of data sharing with a view towards recognition and attribution that are meaningful and appropriate to the medium or discipline concerned and which provide due credit and acknowledgement of all who contributed to the results.
- Extend recognition and attribution both to primary purposes and, as appropriate, to secondary or downstream uses and applications. All parties should act in good faith to ensure that any connections to original sources of data are maintained where appropriate, to the extent permissible by law.

#### Sustainability

- Ensure, where appropriate, the sustainability of the data generated for future use, through both archiving and using appropriate identification and retrieval systems, and through critical appraisal of the mechanisms and systems used for sharing genomic and health-related data.

#### Education and training

- Dedicate education and training resources so as to advance data sharing and data management and to constantly improve data quality and integrity. Education and training resources should lso be dedicated to: fostering and maintaining good records about the effects and impact of data sharing; raising awareness about national health priorities and distribution of health services; building capacity and data sharing infrastructure in countries; and, working towards the building of an evidence base about the advantages and potential limitations of data sharing.

#### Accessibility and dissemination

- Make reasonable efforts to maximize the accessibility of data for research through lawful and proportionate data sharing.
- Promote collaborative partnerships and data sharing that can generate maximum benefit, along with the harmonization of deposit, management and access procedures and use as a means to promote accessibility.
- Seek to make data and research results widely available, including through publication and digital dissemination, whether positive, negative or inconclusive, depending on the nature and use of the data. Dissemination of data and research results should be conducted in a way that both promotes scientific collaboration, reproducibility and broad access to data, and yet minimizes obstacles to data sharing while minimizing harms and maximizing benefits to individuals, families and communities.

## Implementation mechanisms and amendments

1. This Framework should be adopted by organizations and bodies involved in genomic and health-related data sharing. Organizations and bodies adhering to this Framework should take all reasonable and appropriate measures, whether of a regulatory, contractual, administrative or other character, to give effect to the Foundational Principles and Core Elements set out in this Framework in accordance with the international law of human rights and should, by means of all reasonable and appropriate measures, promote their implementation.
2. Any persons, organizations or bodies adhering to this Framework may propose one or more amendments to the present Framework by communicating the amendments to the Regulatory and Ethics Working Group of the Global Alliance for Genomics and Health (the “REWG”). The REWG shall publicly circulate such amendments for comments and possible inclusion in this Framework.
3. The REWG, in collaboration with biomedical, patient advocacy, and ethical and policy organizations and committees, will track the adoption of this Framework and its application through subsequent Policies. It will also routinely review its provisions, be aware of advances in basic research and technology, and ethical and legal developments, and attempt to ensure that this Framework is fit for purpose.

## Appendix 1

### Foundational Human Rights Instruments

* Universal Declaration of Human Rights (UN 1948) (Article 27)

* International Covenant on Economic, Social and Cultural Rights (UN 1966) (Article 15)

### Ethical and Legal Codes and Policies Guiding Data Sharing Behavior

- Constitution of the World Health Organization (WHO 1946)
- Bermuda Principles on Human Genome Sequencing (1996)
- Universal Declaration on the Human Genome and Human Rights (UNESCO 1997)
- Convention on Human Rights and Biomedicine (Council of Europe 1997)
- Statement on DNA Sampling: Control and Access (HUGO 1998)
- Statement on Human Genomic Databases (HUGO Ethics Committee 2002)
- Declaration of Ethical Considerations regarding Health Databases (WMA 2002)
- International Ethical Guidelines for Biomedical Research Involving Human Subjects (CIOMS, WHO 2002)
- Budapest Open Access Initiative (2002)
- Sharing Data from Large-scale Biological Research Projects: A System of Tripartite Responsibility (Fort Lauderdale Statement, 2003)
- International Declaration on Human Genetic Data (UNESCO, IBC 2003)
- European Society of Human Genetics: Data Storage and DNA Banking for Biomedical Research (ESHG 2003)
- Universal Declaration on Bioethics and Human Rights (UNESCO 2005)
- Additional Protocol to the Convention on Human Rights and Biomedicine, concerning Biomedical Research (Council of Europe 2005)
- Recommendation Rec (2006) 4 of the Committee of Ministers to Member States on Research on Biological Materials of Human Origin (Council of Europe 2006)
- OECD Principles and Guidelines for Access to Research Data from Public Funding (OECD 2007)
- International Ethical Guidelines for Epidemiological Studies (CIOMS, WHO 2008)
- Recommendations from the 2008 International Summit on Proteomics Data Release and Sharing Policy (Amsterdam Principles, 2008)
- Guidelines for Human Biobanks and Genetic Research Databases (OECD 2008, 2009)
- Toronto Statement on Prepublication Data Sharing (2009)
- Joint Statement by Funders of Health Research (2011)
- 2012 Best Practices for Repositories: Collection, Storage, Retrieval and Distribution of Biological Material for Research (ISBER 2012)
- Responsible Conduct in the Global Research Enterprise: A Policy Report (InterAcademy Council 2012)
- Declaration of Helsinki (WMA 2013)
- Guidelines governing the Protection of Privacy and Transborder Flows of Personal Data (OECD 2013)

## Appendix 2

Figure 1.

**Figure 1:**
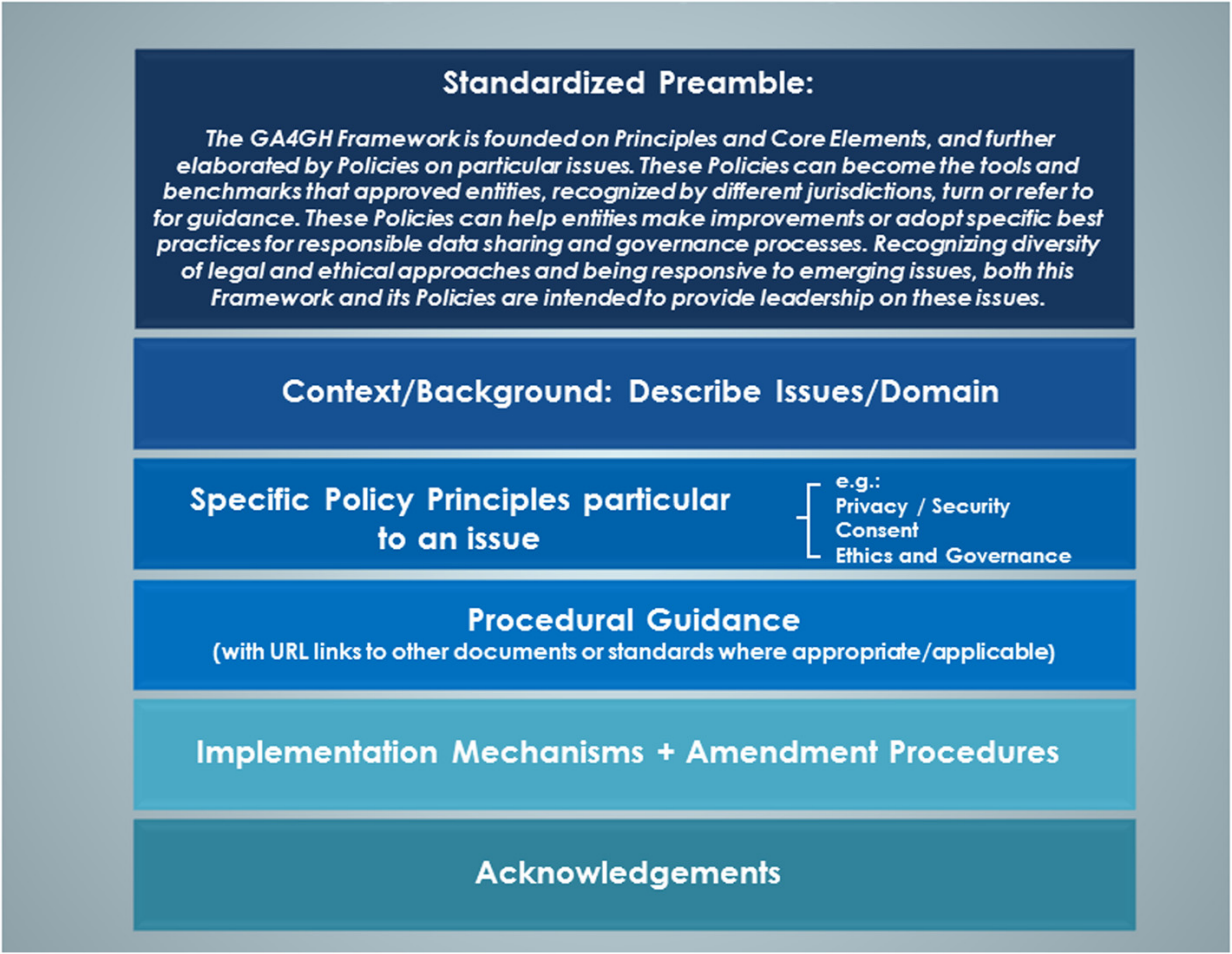
**Global Alliance for Genomics and Health (GA4GH): proposed policy template.**

